# Hepatic artery aneurysm repair: a case report

**DOI:** 10.1186/1752-1947-3-18

**Published:** 2009-01-21

**Authors:** SS Jaunoo, TY Tang, C Uzoigwe, SR Walsh, ME Gaunt

**Affiliations:** 1 Cambridge Vascular Unit, Cambridge University Hospitals NHS Trust Hospital, Cambridge, UK; 2 Department of General Surgery, Royal Shrewsbury Hospital, Mytton Oak Road, Shrewsbury, SY3 8XQ, UK

## Abstract

**Introduction:**

Hepatic artery aneurysms remain a clinically significant entity. Their incidence continues to rise slowly and mortality from spontaneous rupture is high. Repair is recommended in those aneurysms greater than 2 cm in diameter. It is not surprising that vascular comorbidities, such as ischaemic heart disease, are common in surgical patients, particularly those with arterial aneurysms such as these. The decision of when to operate on patients who require urgent surgery despite having recently suffered an acute coronary syndrome remains somewhat of a grey and controversial area. We discuss the role of delayed surgery and postoperative followup of this vascular problem.

**Case presentation:**

A 58-year-old man was admitted with a 5.5 cm hepatic artery aneurysm. The aneurysm was asymptomatic and was an incidental finding as a result of an abdominal computed tomography scan to investigate an episode of haemoptysis (Figure 1). Three weeks prior to admission, the patient had suffered a large inferior myocardial infarction and was treated by thrombolysis and primary coronary angioplasty. Angiographic assessment revealed a large aneurysm of the common hepatic artery involving the origins of the hepatic, gastroduodenal, left and right gastric arteries and the splenic artery (Figures 2 and 3). Endovascular treatment was not considered feasible and immediate surgery was too high-risk in the early post-infarction period. Therefore, surgery was delayed for 3 months when aneurysm repair with reconstruction of the hepatic artery was successfully performed. Graft patency was confirmed with the aid of an abdominal arterial duplex. Plasma levels of conventional liver function enzymes and of alpha-glutathione-*S*-transferase were within normal limits. This was used to assess the extent of any hepatocellular damage perioperatively. The patient made a good recovery and was well at his routine outpatient check-ups.

**Conclusion:**

There is no significant difference in cardiac risk in patients who have undergone vascular surgery within 6 months of a myocardial infarction compared with those who have had the operation in the 6 to12 month time frame. Use of alpha-glutathione-*S*-transferase gives an indication of the immediate state of hepatic function and should be used in addition to traditional liver function tests to monitor hepatic function postoperatively.

## Introduction

Hepatic artery aneurysms (HAAs) are a rare but a clinically important phenomenon. A review of the literature between 1985 and 1995 showed that the HAA had surpassed splenic artery aneurysm (SAA) as the most frequently reported visceral artery aneurysm [1]. This recent trend is thought to be due to the proliferation of centres performing invasive diagnostic and therapeutic hepatobiliary procedures, many of which have hepatic artery pseudo-aneurysm formation as a recognised complication. The natural history of HAA is poorly understood, however, it is suggested that mortality following spontaneous rupture is as high as 40% [2]. Statistics such as these sanction an aggressive approach to the management of the HAA, whenever detected.

It is recommended that aneurysmal repair be considered in HAA larger than 2 cm [2]. In our case, the situation was complicated by the fact that the patient had undergone a large myocardial infarction (MI) 3 weeks prior to admission. The decisions to observe and to operate were both high-risk options. The multidisciplinary team in conjunction with the patient and family had to decide if surgical correction of the HAA was still a viable option and if so, what if any window of the rehabilitation should be allowed before attempting surgical repair.

The collaborative body of the American College of Cardiology (ACC) and the American Heart Association (AHA) stratify vascular surgery as high-risk surgery with a significant probability (> 5%) of a perioperative cardiac event fatal or otherwise. In addition, they recognise a previous history of recent MI (within 3.6 months) in itself as a major predictor of a perioperative cardiac event [3]. The Cleveland Clinic and Bypass Angioplasty Revascularization Investigation (BARI) retrospective studies, however, demonstrated a low perioperative cardiac morbidity in high-risk vascular patients who had previously undergone percutaneous transluminal coronary angioplasty for cardiac indications within 18 months [3]. Back and co-workers [4] in their prospective study similarly observed a reduction in the incidence of perioperative cardiac events in patients with a recent (less than 2 years) history of coronary angioplastic revascularisation.

Current ACC/AHA guidelines suggest that patients who have undergone coronary revascularisation within the last 5 years and have remained asymptomatic are generally a low cardiac risk. In this case, the patient underwent angioplasty and stent insertion. The reduction in perioperative cardiac risk afforded by coronary stenting in patients undergoing vascular surgery has not been fully determined. It is thought to prolong the cardio-protective effect of angioplasty. Very early surgery after coronary stenting or surgery after stenting unsupported by an allied antiplatelet schedule is associated with high incidences of perioperative mortality and morbidity including stent thrombosis, bleeding and embolisation [5,6]. It is suspected that the perioperative cardio-protective effect of coronary stenting is critically dependent upon the patient having completed an antiplatelet regimen parallel to the stenting process. It seems prudent for patients requiring urgent high-risk surgery who have undergone pre-operative coronary stenting to complete their antiplatelet course before surgical intervention is attempted.

In this case, it was decided that a previous MI should not disqualify the patient from vascular repair in light of the high-risk nature and unpredictability of the HAA and the successful coronary revascularisation that had been performed. This though raised the issue of when to schedule surgery. Traditionally vascular surgery has been contraindicated within 6 months of an MI due to inherent cardiac instability leading to high rates of re-infarction and stratospheric concomitant mortality. Such premises are based, however, in old data which predate much of the rapid thrombolysis, cardiac vascularisation and novel cardiotropic therapies currently in routine use. Recent studies have shown no significant difference in cardiac risk in patients who have undergone vascular surgery within 6 months of an MI compared with those who have had the operation in the 6 to 12 month time frame [6].

**Figure 1:**
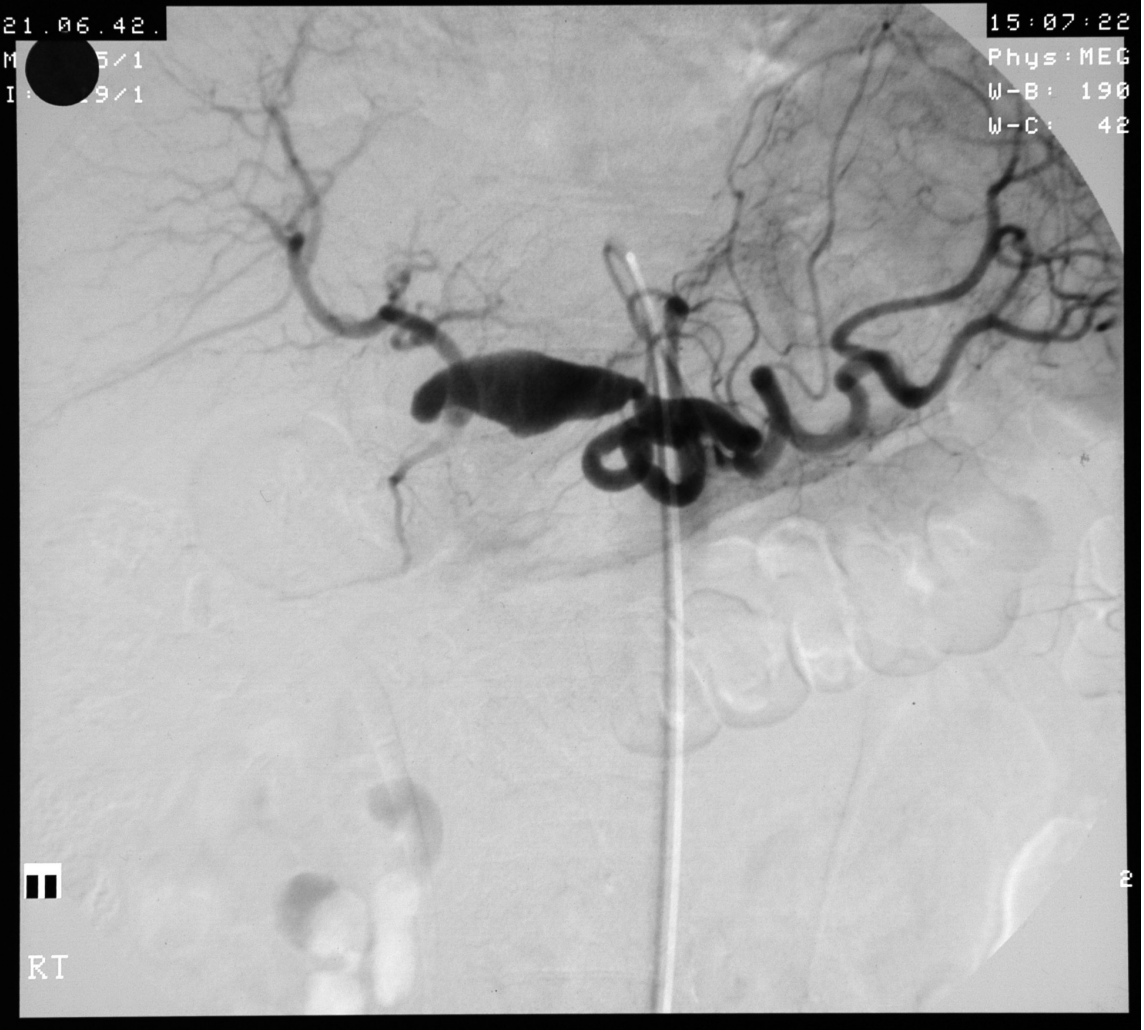
**Angiogram showing the patient's large hepatic artery aneurysm**.

**Figure 2:**
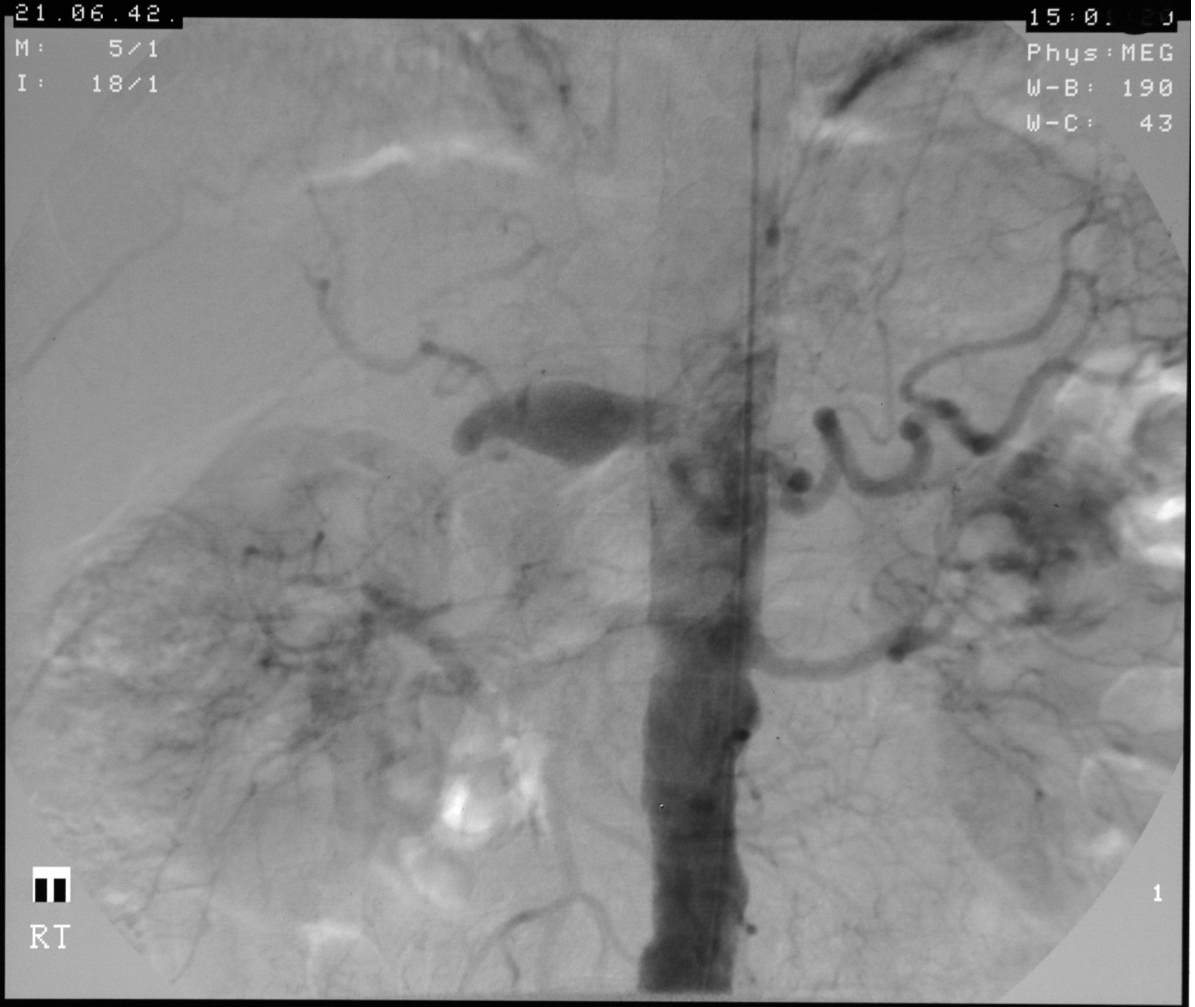
**Angiogram showing the patient's large hepatic artery aneurysm**.

**Figure 3:**
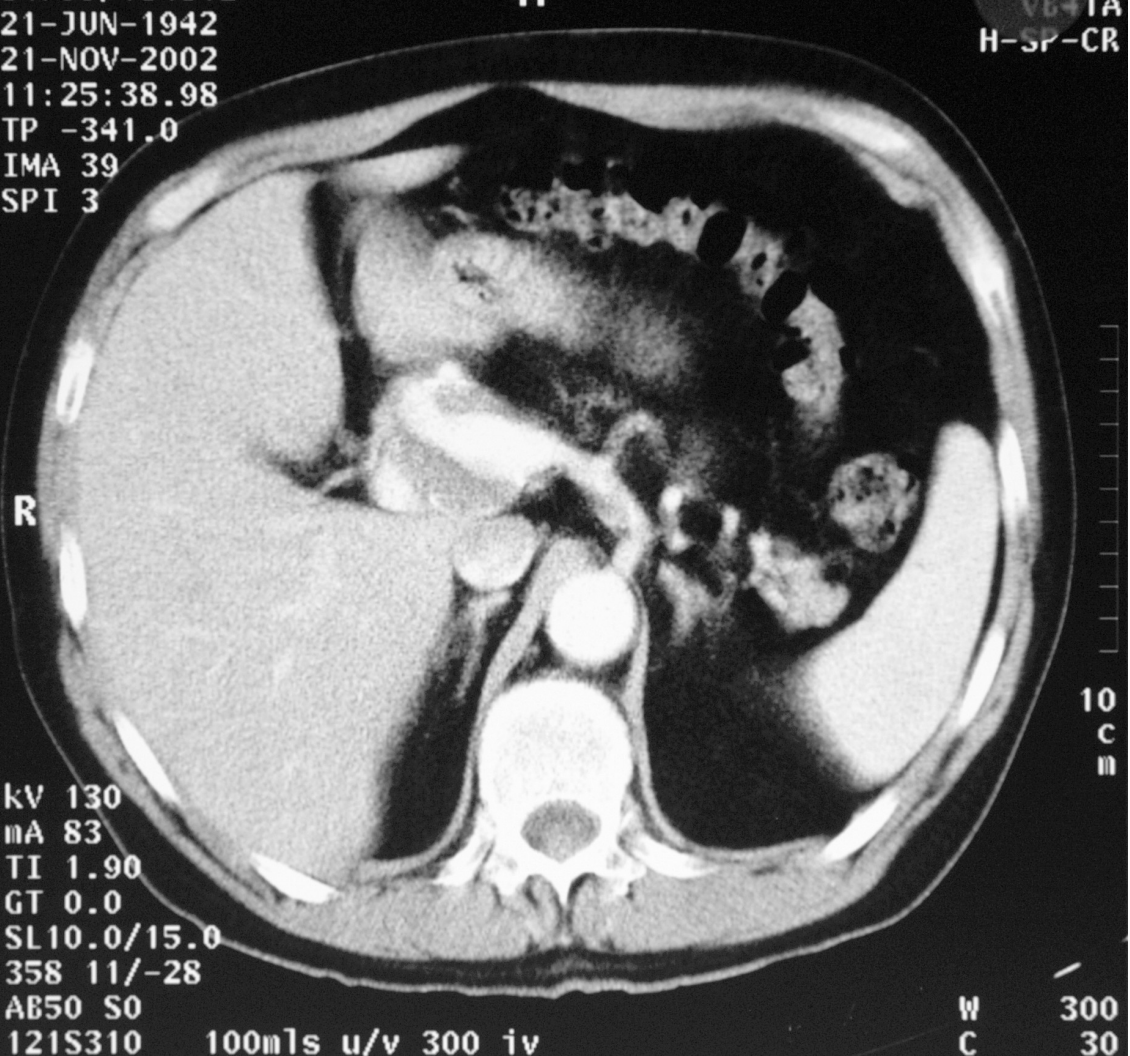
Computed tomography scan showing the hepatic artery aneurysm.

Aneurysms of the hepatic artery propria, namely the section of hepatic artery distal to the junction of the gastroduodenal and right gastric arteries, invariably require vascular reconstruction if ischaemic liver injury is to be avoided. In our patient, arteriotomy was performed with subsequent interposition of reversed a long saphenous vein graft. It is generally suggested, however, that in cases where the gastroduodenal-pancreaticoduodenal artery arcade is patent, then exclusion of the aneurysm is sufficient with no real need for vascular reconstruction. (See figures 1,2 and 3)

This is based on two premises. The first is that the liver receives a significant quantity of blood (70%) from the portal system and secondly, that the anastomotic arcade between the coeliac axis and the superior mesenteric arteries via the right gastric and gastroduodenal arteries should meet any demands above this. Two fundamental principles, one anatomical and the other physiological, have been overlooked. Although the hepatic artery supplies only 30% by volume of blood perfusing the liver, it provides up to 60% of the oxygen metabolised by the organ. This is because, unlike the blood within the portal system, that of the hepatic artery is direct from the aorta and has not bypassed the organs of the gastrointestinal tract. Secondly, the liver is a key homeostatic organ. The liver's blood supply is under extensive but precise endocrine and autonomic regulation such that organ perfusion is closely matched with the organ's metabolic demands. This is achieved by varying the flow through the hepatic artery rather than through the portal vein. It has been shown that if the portal vein is clamped then blood flow through the hepatic artery is increased such that oxygen supply to the liver is almost unchanged. The converse, however, is not true for the portal vein, if the hepatic artery is clamped. Ligation of the common hepatic artery abrogates its regulatory function in hepatic perfusion and thus, in certain physiological situations, oxygen demand may fall short of supply leading to ischaemia. In the postprandial setting, paracrine and endocrine factors cause a global increase in blood flow throughout the coeliac and mesenteric axes, thus ensuring adequate flow through the gastroduodenal-pancreaticoduodenal conduit and adequate perfusion of the liver in preparation for increased metabolic activity following food ingestion.

In situations where the liver is acting in roles divorced from its function as an accessory organ of the gastrointestinal tract, such as during times of fasting or in roles such as gluconeogenesis, protein deamination and metabolism of endocrine and coagulation agents, there is no reactionary increase in the flow through the coeliac-superior mesenteric anastomoses and the liver is susceptible to ischaemic injury if the hepatic artery has been excluded.

Where HAA repair has involved vascular reconstruction, postoperative patency and definitive exclusion of the aneurysm are commonly confirmed with abdominal arterial duplex ultrasound. This alone, however, gives no indication as to the functional status of the liver nor does it alert to any ischaemic injury suffered by the liver perioperatively. In addition, in the long term, if the graft were to undergo progressive stenosis, the resultant effect on the liver would be a crucial piece of information in devising the next stage of the management plan.

We used alpha-glutathione-*S*-transferase (alpha-GST) in conjunction with the conventional liver enzymes to assess the extent of any hepatic ischaemic injury in the immediate and long-term postoperative period. Alpha-GST is a highly useful marker of liver damage. It is found in high concentrations within hepatocytes and is released following hepatic injury and has a short plasma half-life (approximately 90 minutes) [7]. Alpha-GST is superior to the conventional liver functional enzymes as an index of hepatic damage. The conventional hepatic biomarkers have a long plasma half-life. Following hepatic ischaemic injury, they remain elevated after alpha-GST has returned to within normal limits. The short plasma half-life of alpha-GST enables it to reliably reflect changes in hepatocellular status in the postoperative period. It is being increasingly used in the transplant patient to assess the degree of hepatic injury post-transplantation secondary to either ischaemia or rejection. One trial observed that monitoring alpha-GST in the transplant patient resulted in improved patient care with an increase in graft survival, a fall in mortality and morbidity and a lower frequency of postoperative invasive diagnostic procedures up to 100 days postoperatively [8].

## Conclusion

Traditionally, vascular surgery was contraindicated within 6 months of an acute coronary syndrome due to the high risk of re-infarction and its associated mortality. However, these assumptions were based upon studies conducted before the introduction of clinical interventions such as rapid thrombolysis, cardiac revascularisation and new cardiotropic therapies which are routinely used nowadays. More up-to-date studies have demonstrated that there is no significant difference in cardiac risk in those patients who have undergone vascular surgery within 6 months of an MI.

In the long term, we intend to use both alpha-GST in addition to traditional hepatic biomarkers to assess postoperative hepatic function and progress. The former gives an indication of the immediate state of hepatic function, while the latter reflects the long-term condition of the liver. To the best of our knowledge, we report the first use of this biochemical marker in the setting of visceral artery reconstruction.

## Abbreviations

AC: American College of Cardiology; AHA: American Heart Association; alpha-GST: alpha-glutathione-*S*-transferase; HAA: hepatic artery aneurysms; MI: myocardial infarction; SAA: splenic artery aneurysm.

## Consent

Written informed consent was obtained from the patient for publication of this case report and any accompanying images. A copy of the written consent is available for review by the Editor-in-Chief of this journal.

## Competing interests

The authors declare that they have no competing interests.

## Authors' contributions

Conception and design: TYT, CU, MEG. Data collection: CU, TYT, SRW, MEG. Writing the article: SSJ, CU, TYT, SRW, MEG. Critical revision of the article: SSJ, TYT, MEG. Final approval of the article: SSJ, TYT, CU, SRW, MEG. Overall responsibility: MEG
